# A nomogram to predict the risk of colorectal anastomotic leakage combining inflammatory-nutritional and abdominal aorta calcium index

**DOI:** 10.3389/fsurg.2022.1008448

**Published:** 2023-01-06

**Authors:** Zhaoxiong Zhang, Weilin Sun, Jun Wang, Yuanlin Deng, Yongjia Yan, Dong Li, Weihua Fu

**Affiliations:** ^1^ Department of General Surgery, Tianjin Medical University General Hospital, Tianjin, China; ^2^ Department of Gastrointestinal Surgery, The People's Hospital of Qiannan, Duyun, China; ^3^ Department of Radiology, Tianjin Medical University General Hospital, Tianjin, China

**Keywords:** arterial calcification, anastomotic fistula, colorectal cancer, inflammation, nutrition

## Abstract

**Background:**

Anastomotic leakage is a serious complication after colorectal cancer surgery, which affects the quality of life and the prognosis. This study aims to create a novel nomogram to predict the risk of anastomotic leakage for patients with colorectal cancer based on the preoperative inflammatory-nutritional index and abdominal aorta calcium index.

**Methods:**

292 patients at Tianjin Medical University General Hospital (Tianjin, China) from January 2018 to October 2021 who underwent colorectal cancer surgery with a primary anastomosis were retrospectively reviewed. A nomogram was constructed based on the results of multivariate logistic regression model. The calibration curves and receiver operating characteristic curves were used to verify the efficacy of the nomogram.

**Results:**

Univariate and multivariate analyses showed that tumor location (*P* = 0.002), preoperative albumin (*P* = 0.006), preoperative lymphocyte (*P* = 0.035), preoperative neutrophil to lymphocyte ratio (*P* = 0.024), and superior mesenteric artery calcium volumes score (*P* = 0.004) were identified as the independent risk factors for postoperative anastomotic leakage in patients with colorectal carcinoma. A nomogram was constructed based on the results of the multivariate analysis, and the C-index of the calibration curves was 0.913 (95%CI: 0.870–0.957) in the training cohort and 0.840 (95%CI: 0.753–0.927) in the validation cohort.

**Conclusion:**

The nomogram, combining basic variables, inflammatory-nutritional index and abdominal aorta calcium index, could effectively predict the possibility of postoperative anastomotic leakage for patients with colorectal cancer, which could guide surgeons to carry out the appropriate treatment for the prevention of anastomotic leakage.

## Introduction

With the progress of surgical technology and oncology surgery, surgical treatment has become the principal treatment for the patients with resectable colorectal cancer, which greatly improves their survival prognosis (1, 2). However, postoperative complications, which affect the recovery of patients after surgery, and even threaten the survival of patients, have always been an urgent problem that plagues surgeons. Among postoperative complications, anastomotic leakage (AL) is one of the most common and serious postoperative complications, with an incidence of 4%–29% (3) AL not only prolongs the patient's hospital stay, reduces the patient's quality of life, but even affects the patient's survival after surgery (4–6). Surgeons have made much effort to avoid AL in clinical practice, such as prophylactic ileostomy, and intraoperative evaluation of blood flow with indocyanine green (7–9) However, there is still no objective method for helping surgeons to predict the occurrence of AL in patients with colorectal cancer before surgery.

At present, while the recognized factors related to AL after colorectal cancer surgery were not clear, we could confirm that AL is the result of multiple factors (10, 11). Some studies showed that the tumor located in the rectum, preoperative malnutrition, excessive inflammatory response and other factors could be related to postoperative AL (10, 12–14). Patients with gastrointestinal cancer usually had a higher risk of preoperative malnutrition, which might be associated with poor postoperative outcomes (14, 15). Several studies showed that adequate nutritional support might improve this situation for patients with high nutritional risk (16). The nutritional status of the patients seemed to be an important factor for clinicians to predict the early prognosis. In recent years, numerous studies confirmed that cancer-associated inflammation was an important factor to produce tumor-promoting effects (17). Preoperative systemic inflammation of colorectal cancer patients was an important factor associated with the poor prognosis and high morbidity of AL (10, 18). The results of hematological tests were great indicators of preoperative systemic inflammation in patients with colorectal cancer, and the relevant data was readily available in almost all patients. Moreover, abdominal aorta calcification, which could damage the vascular function and might reduce anastomotic blood flow, might increase the risk of colorectal AL (19, 20). Therefore, accurate preoperative assessment of the above risk factors was essential to prevent AL.

This study aimed to establish a model that could predict the probability of postoperative AL by assessing the preoperative status of patients with colorectal cancer. In this study, the index of nutrition, the index of inflammation and the artery calcium volumes score were considered as the risk factors for AL, and a better nomogram was prepared to guide clinicians in decision-making.

## Patients and methods

### Patients

Between January 2018 and October 2021, a total of 327 patients underwent colorectal cancer resection at Tianjin Medical University General Hospital. The clinicopathologic data of these patients were retrospectively reviewed from the hospital information system after receiving Institutional Review Board approval. The data included basic variables (gender, age, alcohol, smoke, diabetes, BMI, tumor location, Obstruction, ASA score and pTNM stage), preoperative nutritional variables (hemoglobin, albumin, and prognostic nutritional index), inflammatory variables (WBC, neutrophil, lymphocyte, platelet, neutrophil-lymphocyte ratio; and platelet-lymphocyte ratio), and vascular condition variables (superior mesenteric artery calcium volumes score, inferior mesenteric artery calcium volumes score, and abdominal aorta calcium volumes score). Eligibility criteria included: (I) proven histologically primary colorectal carcinoma; (II) R0 colorectal cancer surgery with D3 lymph node dissection and a primary anastomosis; (III) complete and available clinicopathological data. The exclusion criteria were: (I) less than 18 years old; (II) history of neoadjuvant chemotherapy or radiotherapy; (III) the patients who had surgery for other cancers or bowel resection for any reason.

The authors are accountable for all aspects of the work in ensuring that questions related to the accuracy or integrity of any part of the work are appropriately investigated and resolved.

### Surgical procedure

All patients underwent open or laparoscopic radical surgery for colorectal cancer. The linear stapler device was applied in the intestinal anastomosis of colon cancer patients, and the circular stapler device was applied in the anastomosis of rectal cancer patients. Then we performed the seromuscular suturing with a 3–0 absorbable suture to reduce the intestinal tension and ensure the mechanical integrity of the anastomosis for colon cancer surgery. And the air-leak test was used to confirm mechanical integrity after intestinal anastomosis in rectal cancer surgery.

### Evaluation of Al

AL was diagnosed by the presence of gas or intestinal contents drained from the wound or drainage tube, the evidence of the intraperitoneal infection, or the positive signs of CT or endoscopy. Digital rectal examination was also used to identify AL in patients with rectal cancer.

### Preoperative variables and vascular evaluation

**Figure 1:**
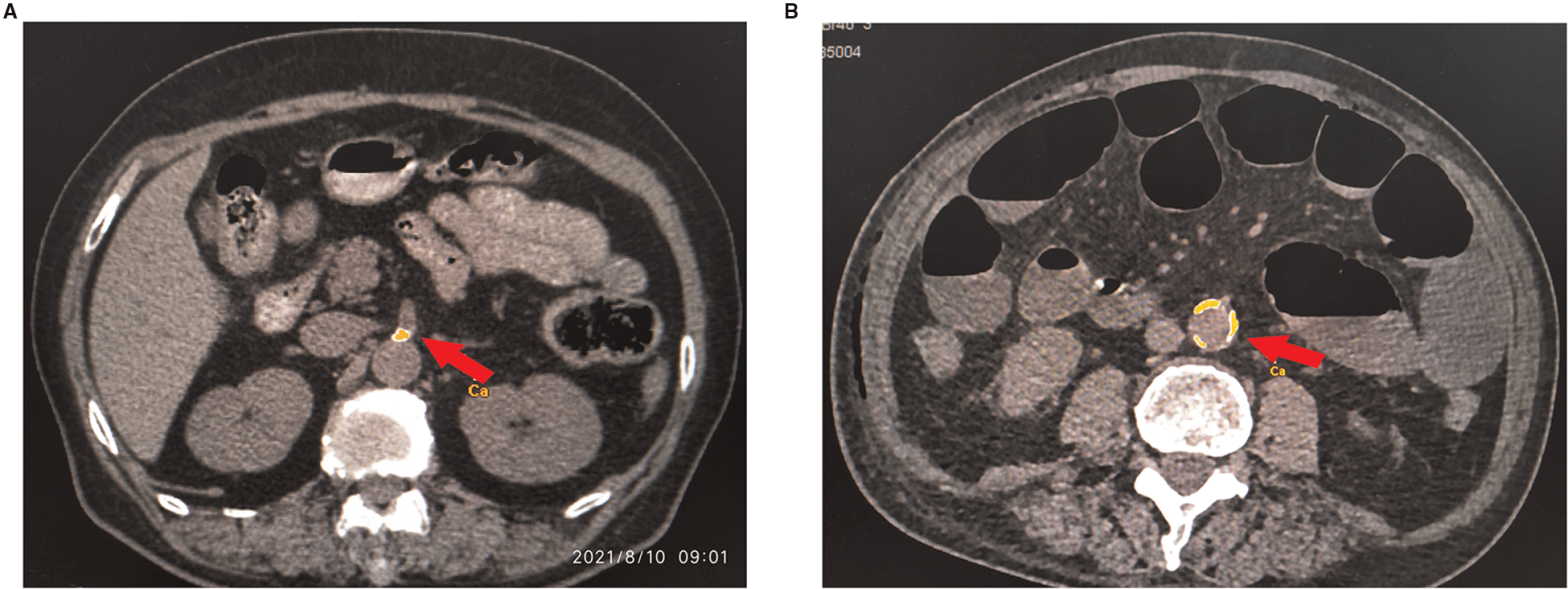
The MDCT images of abdominal aorta, SMA and IMA calcification. The arrow in the images indicates the site of arterial calcification. The plaque was outlined, and then its area was obtained by the software (Syngo Calcium Scoring, Siemens). (**A**) The calcification at the origin of SMA on the aorta. (**B**) The calcification of the abdominal aorta.

We recorded the results of the preoperative laboratory examination 7 days before the operation. The pathological TNM staging was assessed according to the 8th editions of the American Joint Committee on Cancer (AJCC) staging system. Prognostic nutritional index (PNI) values were calculated with the formula: 10 × albumin (g/L) + 0.005 × total lymphocyte count. Neutrophil-lymphocyte ratio (NLR) value was calculated as neutrophils count divided by absolute lymphocyte count. Platelet-lymphocyte ratio (PLR) value was calculated as platelet count divided by absolute lymphocyte count. The vascular condition was evaluated through a preoperative multi-detector computed tomography (MDCT) image. The MDCT images of aortic between the origin of the superior mesenteric artery (SMA) and inferior mesenteric artery (IMA) were analyzed on the software (Syngo Calcium Scoring, Siemens). The plaque area in the aortic lumen, which was more hyperdense than the vascular lumen and adjacent parenchyma (density more than 130 HU), was considered as vascular calcification (21). The artery calcium volume score was measured by calculating the plaque area multiplied by the slice spacing. The SMA and IMA calcium volume score was determined by calcium volume scores at the origin of SMA and IMA on the aorta, respectively. In this study, the abdominal aorta calcium volume score was calculated as the total score of SMA and IMA calcium volume (Figure 1).

Patients were divided into a low or high group according to the cut-off value of albumin, WBC, neutrophil, lymphocyte, platelet, NLR, PLR, PNI, SMA calcium volume score, IMA calcium volume score and abdominal aorta calcium volume score, which was calculated through receiver operating characteristic (ROC) curve analysis.

### Nomogram model

First, the univariate and multivariate analyses were performed to identify the independent risk factors for AL after colorectal cancer surgery. Then, the nomogram was created based on the results of the multivariate logistic regression analysis. The nomogram was subjected to 1,000 bootstrap resamples to calculate the concordance index (c-index) which could be used to estimate the predictive accuracy of the model (22). And the calibration curves were created to graphically present the relationship between the observed results and the predicted probabilities.

### Statistical analysis

The *χ*^2^ or Fisher's exact test was used for categorical variables, and the *t*-test was used for continuous variables. Factors that showed significant differences in the univariate analysis (*P* < 0.05) were included in the multivariate analysis. Multivariate analysis was performed using a logistic regression model for the evaluation of the predictive risk factors, in which, the odds ratios (ORs) and 95% confidence interval (CI) were calculated. In all the other statistical analyses, significance was defined as *P* < 0.05 (two-sided). ROC analysis was performed to compare the accuracy of the nomogram and five independent risk factors and area under the curve (AUC) were also calculated. All statistical analyses were performed using the statistical analysis program package IBM SPSS Statistics (Version 24.0; IBM Corp., New York, USA, RRID: SCR_019096). And R 4.0.1 software (RMS, riskRegression and pROC packages; Institute for Statistics and Mathematics, Vienna, Austria; http://www.r-project.org/, RRID: SCR_001905).

## Results

### Clinical characteristics of patients

**Figure 2:**
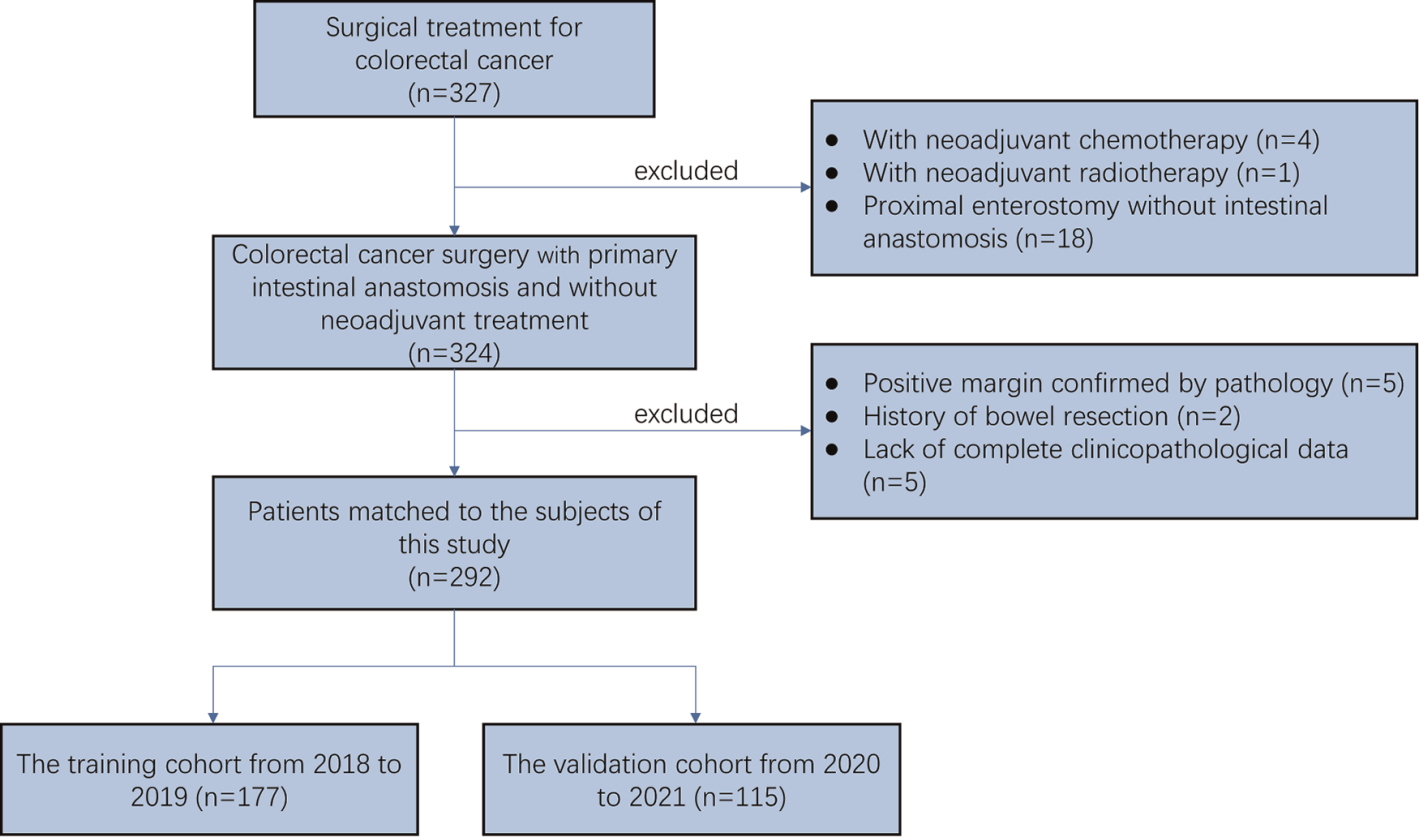
The flow chart of the selection process for the colorectal cancer patients from from January 2018 to October 2021.

According to the above eligibility and exclusion criteria, 292 patients in total were included in this study. These patients were classified into training cohort (date of surgery: 2018–2019, *n* = 177) and validation cohort (date of surgery: 2020–2021, *n* = 115) according to the date of surgery (Figure 2). The clinical and pathologic characteristics of 292 patients in this retrospective study were listed in Table 1. These patients included 168 males and 124 females. The median age was 67 (range 26–90). A total of 87 (29.79%) patients had a history of alcohol, and 94 (32.19%) had a history of smoking. 66 (22.60%) patients were diagnosed of diabetes. The median BMI was 23.73 (range 14.61–34.19). Tumors were distributed on right colon (*n* = 121, 41.44%), transverse colon (*n* = 12, 4.11%), left colon (*n* = 25, 8.56%), sigmoid colon (*n* = 58, 19.86%) and rectum (*n* = 76, 26.03%). A total of 37 (12.67%) patients had AL, and the number of patients with AL was 6 in the right-sided colon, 3 in the transverse colon, 3 in the left-sided colon, 9 in the sigmoid colon and 16 in the rectum. 49 (16.78%) patients had preoperative obstruction and the ASA score of 135 (46.23%) patients is 3. The pT stage of tumor for most patients was pT4a (203, 69.52%), and most patients had no lymph node metastasis (N0: 178, 60.96%). The results of hematological tests were presented in the form of the median (range) and mean (SD). Then, the Clinical characteristics between the training and validation cohort were estimated and no significant differences were shown, which was presented in Table 2.

**Table 1:** Clinicopathologic characteristics of all patients.

Characteristics		All patients (*n* = 292)
Gender [No. (%)]		
Male	168	(57.53%)
Female	124	(42.47%)
Age [No. (%)]		
<60 years	57	(19.52%)
≥60 years	235	(80.48%)
Alcohol [No. (%)]		
No	205	(70.21%)
Yes	87	(29.79%)
Smoke [No. (%)]		
No	198	(67.81%)
Yes	94	(32.19%)
Diabetes [No. (%)]		
No	226	(77.40%)
Yes	66	(22.60%)
BMI [No. (%)]		
<18.5 kg/m^2^	23	(7.88%)
18.5–24 kg/m^2^	138	(47.26%)
≥24 kg/m^2^	131	(44.86%)
Tumor location [No. (%)]		
Right-sided colon	121	(41.44%)
Transverse colon	12	(4.11%)
Left-sided colon	25	(8.56%)
Sigmoid colon	58	(19.86%)
Rectum	76	(26.03%)
Obstruction [No. (%)]		
No	243	(83.22%)
Yes	49	(16.78%)
ASA score [No. (%)]		
≤2	157	(53.77%)
3	135	(46.23%)
pT stage [No. (%)]		
pTis	12	(4.11%)
pTI	16	(5.48%)
pTII	32	(10.96%)
pT4a	203	(69.52%)
pT4b	29	(9.93%)
pN stage [No. (%)]		
pN0	178	(60.96%)
pN1a	32	(10.96%)
pN1b	42	(14.38%)
pN2a	22	(7.53%)
pN2b	18	(6.16%)
pM stage [No. (%)]		
pM0	279	(95.55%)
pM1	13	(4.45%)
Preoperative hemoglobin [No. (%)]		
<110 g/L	114	(39.04%)
≥110 g/L	178	(60.96%)
Preoperative albumin (g/L)		
Median (range)	37	(27–51)
Mean (SD)	37.15	(3.80)
Preoperative WBC (×10^9^/L)		
Median (range)	6.24	(2.55–16.47)
Mean (SD)	6.55	(2.04)
Preoperative neutrophil (×10^9^/L)		
Median (range)	3.66	(1.09–12.88)
Mean (SD)	4.03	(1.87)
Preoperative lymphocyte (×10^9^/L)		
Median (range)	1.64	(0.3–4.15)
Mean (SD)	1.71	(0.64)
Preoperative platelet (×10^9^/L)		
Median (range)	268.00	(106–717)
Mean (SD)	282.87	(100.42)
Preoperative NLR		
Median (range)	2.17	(0.47–24.30)
Mean (SD)	2.95	(2.98)
Preoperative PLR		
Median (range)	162.43	(53.58–750.00)
Mean (SD)	190.77	(115.05)
Preoperative PNI		
Median (range)	45.25	(30.35–62.05)
Mean (SD)	45.72	(5.19)
SMA calcium volumes core (mm^3^)		
Median (range)	0.00	(0–1514.50)
Mean (SD)	51.62	(164.72)
IMA calcium volumes core (mm^3^)		
Median (range)	25.95	(0–3145.40)
Mean (SD)	174.78	(390.63)
abdominal aorta calcium volumes core (mm^3^)		
Median (range)	39.75	(0–4659.90)
Mean (SD)	226.40	(539.13)
Anastomotic leakage [No. (%)]		
No	255	(87.33%)
Yes	37	(12.67%)

Note: WBC, white blood cell; NLR, neutrophil-lymphocyte ratio; PLR, platelet-lymphocyte ratio; PNI, prognostic nutritional index; SMA, superior mesenteric artery; IMA, inferior mesenteric artery; SD, standard deviation.

**Table 2:** Comparison of patients in the training and validation cohorts.

Characteristics	Training cohort (*n* = 177)	Validation cohort (*n* = 115)	*P*
Gender (No.)			
Male	105	63	0.443
Female	72	52	
Age (No.)			
<60 years	35	22	0.892
≥60 years	142	93	
Alcohol (No.)			
No	125	80	0.847
Yes	52	35	
Smoke (No.)			
No	118	80	0.604
Yes	59	35	
Diabetes (No.)			
No	135	91	0.568
Yes	42	24	
BMI (No.)			
<18.5 kg/m^2^	12	11	0.554
18.5–24 kg/m^2^	82	56	
≥24 kg/m^2^	83	48	
Tumor location (No.)			
Right-sided colon	84	37	0.067
Transverse colon	8	4	
Left-sided colon	11	14	
Sigmoid colon	31	27	
Rectum	43	33	
Obstruction (No.)			
No	148	95	0.822
Yes	29	20	
ASA score (No.)			
≤2	97	60	0.660
3	80	55	
pT stage (No.)			
pTis	8	4	0.114
pTI	8	8	
pTII	19	13	
pT4a	118	85	
pT4b	24	5	
pN stage (No.)			
pN0	103	75	0.431
pN1a	20	12	
pN1b	28	14	
pN2a	12	10	
pN2b	14	4	
pM stage (No.)			
pM0	168	111	0.515
pM1	9	4	
Preoperative hemoglobin (No.)			
<110 g/L	72	42	0.477
≥110 g/L	105	73	
Preoperative albumin (No.)			
<37.5 g/L	89	65	0.297
≥37.5 g/L	88	50	
Preoperative WBC (No.)			
<6.5 × 10^9^/L	94	67	0.387
≥6.5 × 10^9^/L	83	48	
Preoperative neutrophil (No.)			
<2.6 × 10^9^/L	34	20	0.696
≥2.6 × 10^9^/L	143	95	
Preoperative lymphocyte (No.)			
<1.35 × 10^9^/L	45	41	0.061
≥1.35 × 10^9^/L	132	74	
Preoperative platelet (No.)			
<270.5 × 10^9^/L	92	64	0.539
≥270.5 × 10^9^/L	85	51	
Preoperative NLR (No.)			
<1.465	47	22	0.145
≥1.465	130	93	
Preoperative PLR (No.)			
<195.715	121	73	0.388
≥195.715	56	42	
Preoperative PNI (No.)			
<41.825	35	25	0.685
≥41.825	142	90	
SMA calcium volumes core (No.)			
<3.2 mm^3^	111	74	0.777
≥3.2 mm^3^	66	41	
IMA calcium volumes core (No.)			
<19.7 mm^3^	85	50	0.447
≥19.7 mm^3^	92	65	
Abdominal aorta calcium volumes core (No.)			
<19.6 mm^3^	84	50	0.505
≥19.6 mm^3^	93	65	
Anastomotic leakage (No.)			
No	156	99	0.607
Yes	21	16	

Note: WBC, white blood cell; NLR, neutrophil-lymphocyte ratio; PLR, platelet-lymphocyte ratio; PNI, prognostic nutritional index; SMA, superior mesenteric artery; IMA, inferior mesenteric artery.

We chose the cutoff value of the albumin, WBC, neutrophil, lymphocyte, platelet, NLR, PLR, PNI, SMA calcium volumes score, IMA calcium volumes score and abdominal aorta calcium volumes score to divide the patients into low and high groups, according to ROC curves.

### Correlation analysis of risk factors for postoperative Al

**Figure 3:**
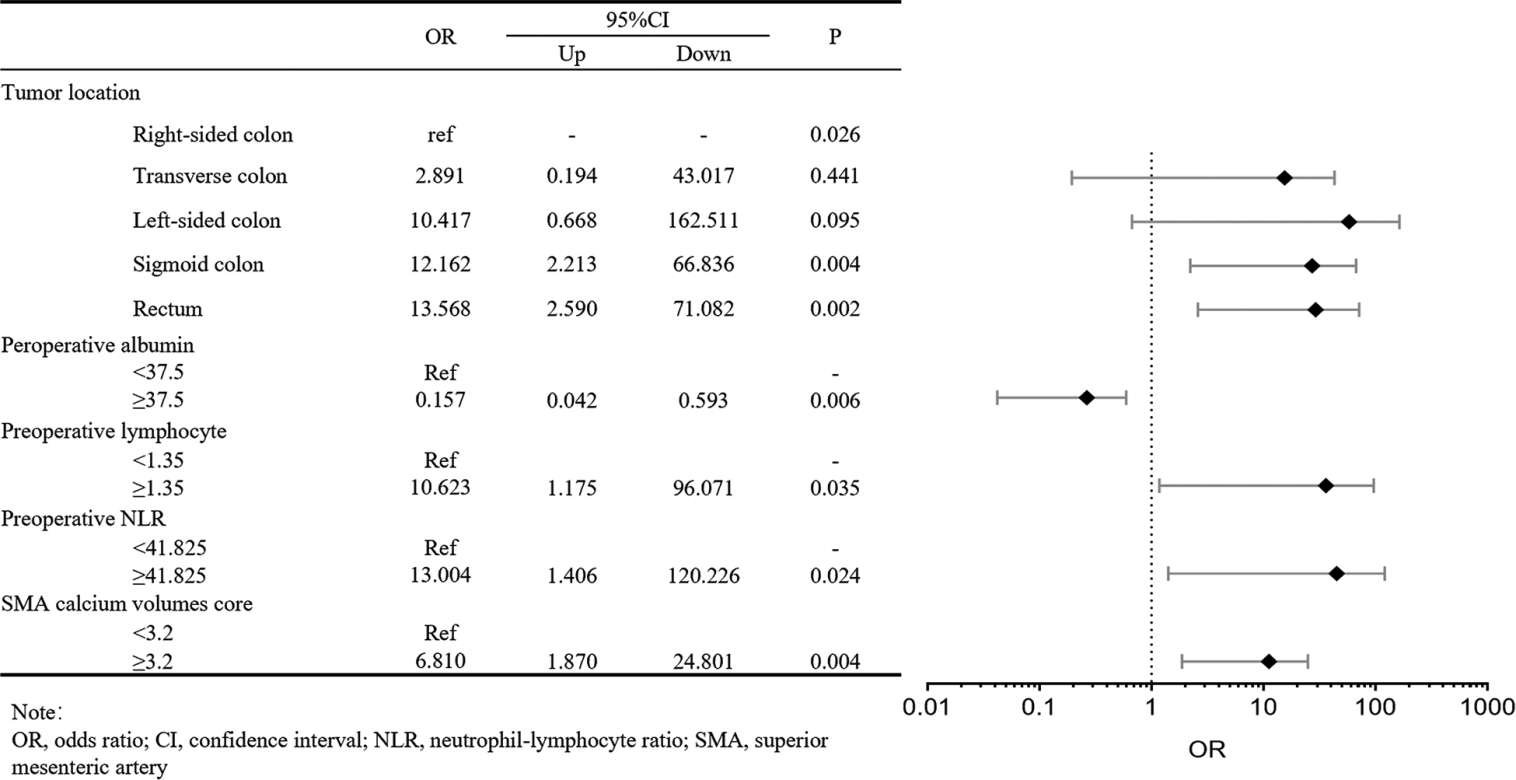
Forest plot about multivariate logistic regression analysis of clinicopathologic characteristics in the training cohort for anastomotic leakage.

We investigated the potential risk factors for AL by univariate analysis in the training cohort (Table 3). The univariate analysis showed the tumor located in the sigmoid colon and rectum (*P* = 0.007), low preoperative albumin (*P* = 0.011), high preoperative lymphocyte (*P* = 0.021), high preoperative NLR (*P* = 0.016), high SMA calcium volumes score (*P* = 0.001), high IMA calcium volumes score (*P* = 0.001) and high abdominal aorta calcium volumes score (*P* = 0.001) were significantly correlated with the occurrence of postoperative AL. Then, these significant variables were performed in the forward step procedures by the multivariate logistics regression model. We observed tumor location (transverse colon, OR: 2.891, 95% CI: 0.194–43.017, *P* = 0.441; left colon, OR: 10.417, 95% CI: 0.668–162.511, *P* = 0.095; sigmoid colon, OR: 12.162, 95% CI: 2.213–66.836, *P* = 0.004; rectum, OR: 13.568, 95% CI: 2.590–71.082, *P* = 0.002), preoperative albumin (OR: 0.157, 95% CI: 0.042–0.593, *P* = 0.006), preoperative lymphocyte (OR: 10.623, 95% CI: 1.175–96.071, *P* = 0.035), preoperative NLR (OR:13.004, 95% CI: 1.406–120.226, *P* = 0.024), SMA calcium volumes score (OR: 6.810, 95% CI: 1.870–24.801, *P* = 0.004) were identified as the independent risk factors for postoperative AL in patients with colorectal carcinoma (Figure 3).

**Table 3:** Univariate analysis of risk factors for anastomotic leakage in training cohort.

Characteristics	Anastomotic leakage		*P*
	No (*n* = 156)	Yes (*n* = 21)	
Gender (No.)			
Male	95	10	0.245
Female	61	11	
Age (No.)			
<60 years	29	6	0.432^†^
≥60 years	127	15	
Sake index	46.1 ± 132.9	38.6 ± 74.9	0.067
Brinkman index	183.1 ± 394.5	178.1 ± 286.9	0.329
Diabetes (No.)			
No	122	13	0.169^†^
Yes	34	8	
BMI (No.)			
<18.5 kg/m^2^	11	1	0.599
18.5–24 kg/m^2^	74	8	
≥24 kg/m^2^	71	12	
Tumor location (No.)			
Right-sided colon	80	3	0.007^*‡^
Transverse colon	7	1	
Left-sided colon	10	1	
Sigmoid colon	25	7	
Rectum	34	9	
Obstruction (No.)			
No	132	16	0.506^†^
Yes	24	5	
ASA score (No.)			
≤2	87	10	0.481
3	69	11	
pT stage (No.)			
pTis	8	0	0.509^‡^
pTI	6	2	
pTII	17	2	
pT4a	105	13	
pT4b	20	4	
pN stage (No.)			
pN0	91	12	0.734^‡^
pN1a	17	3	
pN1b	24	4	
pN2a	12	0	
pN2b	12	2	
pM stage (No.)			
pM0	149	19	0.647^†^
pM1	7	2	
Preoperative hemoglobin (No.)			
<110 g/L	63	9	0.829
≥110 g/L	93	12	
Preoperative albumin (No.)			
<37.5 g/L	73	16	0.011*
≥37.5 g/L	83	5	
Preoperative WBC (No.)			
<6.5 × 10^9^/L	87	7	0.053
≥6.5 × 10^9^/L	69	14	
Preoperative neutrophil (No.)			
<2.6 × 10^9^/L	34	1	0.122^†^
≥2.6 × 10^9^/L	122	20	
Preoperative lymphocyte (No.)			
<1.35 × 10^9^/L	44	1	0.021*
≥1.35 × 10^9^/L	112	20	
Preoperative platelet (No.)			
<270.5 × 10^9^/L	86	10	0.517
≥270.5 × 10^9^/L	70	11	
Preoperative NLR (No.)			
<1.465	46	1	0.016*
≥1.465	110	20	
Preoperative PLR (No.)			
<195.715	103	18	0.069
≥195.715	53	3	
Preoperative PNI (No.)			
<41.825	28	7	0.171^†^
≥41.825	128	14	
SMA calcium volumes core (No.)			
<3.2 mm^3^	105	6	0.001*
≥3.2 mm^3^	51	15	
IMA calcium volumes core (No.)			
<19.7 mm^3^	82	3	0.001*
≥19.7 mm^3^	74	18	
Abdominal aorta calcium volumes core (No.)			
<19.6 mm^3^	81	3	0.001*
≥19.6 mm^3^	75	18	

Note: WBC, white blood cell; Brinkman index: Number of cigarettes per day multiplied by years of smoking; Sake index: weight (g)/22 of ethanol consumed per day multiplied by years of drinking; NLR, neutrophil-lymphocyte ratio; PLR, platelet-lymphocyte ratio; PNI, prognostic nutritional index; SMA, superior mesenteric artery; IMA, inferior mesenteric artery.

* *P* < 0.05.

^†^ Continuity correction.

^‡^ Fisher's exact test.

### Creation and confirmation of the nomogram

**Figure 4:**
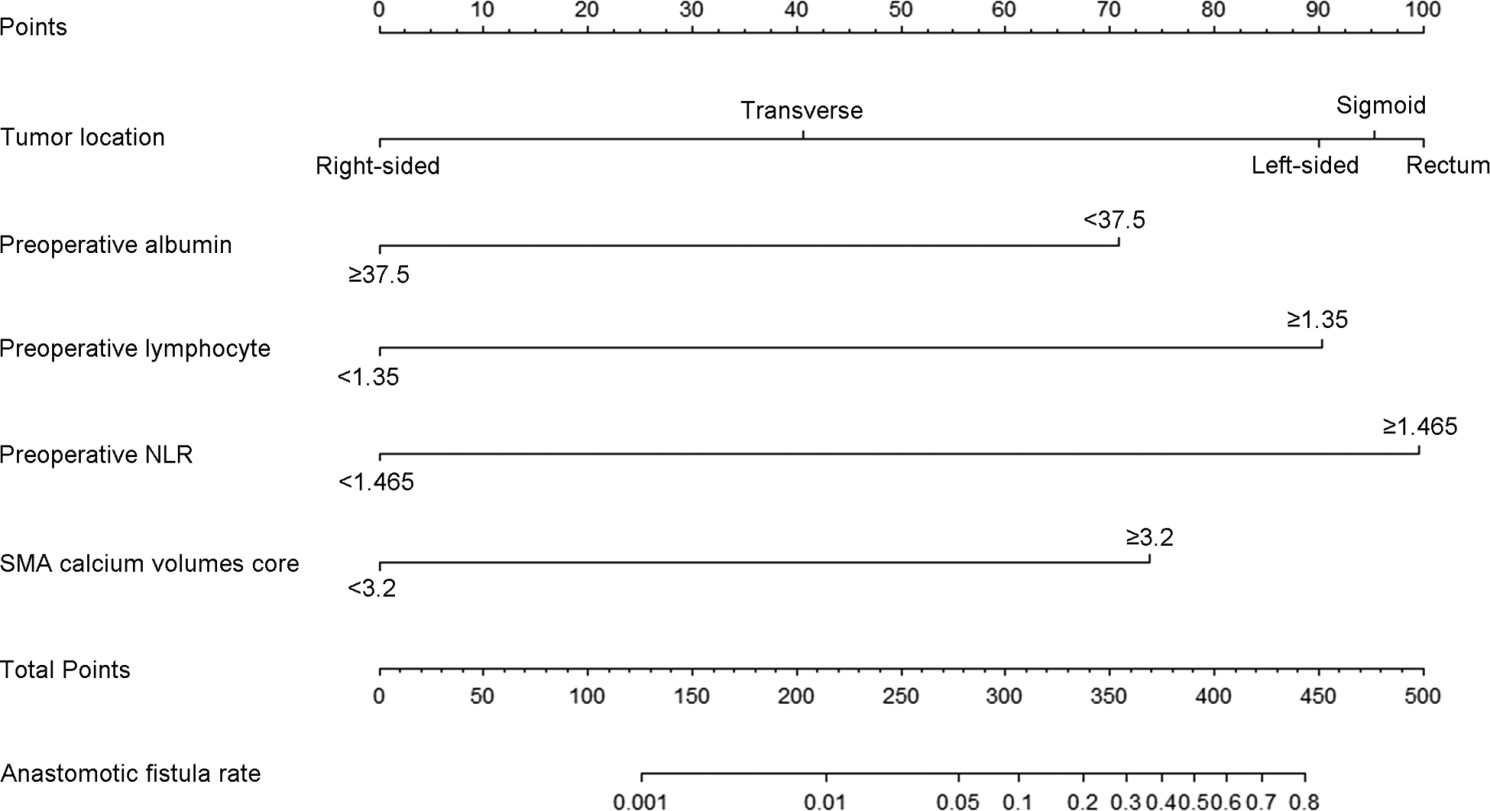
The nomogram predicting postoperative AL in patients with colorectal adenocarcinoma. Match the characteristics of patients to the scale of each risk factor in the nomogram to get the corresponding points. All points were added up to obtain the total points, and then total points was matched to the scale of anastomotic fistula rate to obtain the probability of AL. NLR, neutrophil-lymphocyte ratio.

Based on the above results of univariate and multivariate analysis, we chose the tumor location, preoperative albumin, preoperative lymphocyte, preoperative NLR, and SMA calcium volumes score as factors to create a nomogram for predicting postoperative AL in patients with colorectal carcinoma. The occurrence probability of postoperative AL can be predicted by calculating the points of each variate and projecting the total points to the bottom scale (Figure 4).

**Figure 5:**
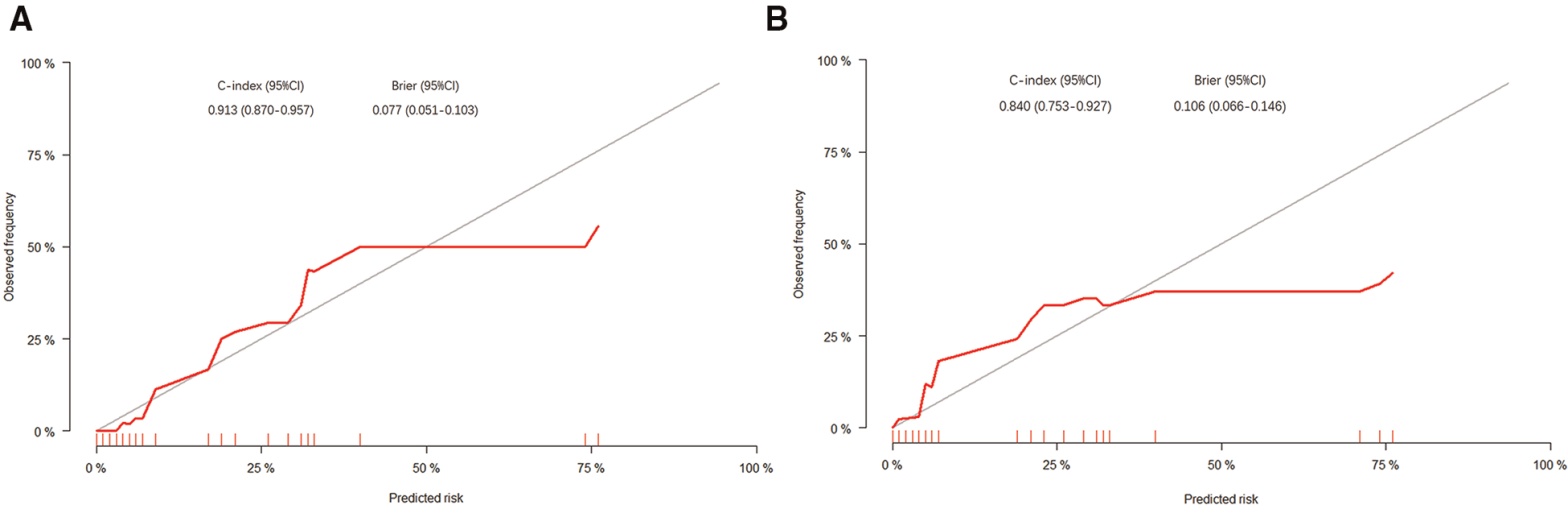
The calibration curves of the nomogram in the training cohort (**A**) and validation cohort (**B**). The *X*-axis represented the possibility of AL predicted by the nomogram, and the *Y*-axis represented the possibility of AL observed in patients.

Then, in order to evaluate the predictive ability of the nomogram model, we performed 1,000 resampling bootstrap analyses in both the training cohort and validation cohort. And the calibration curves were illustrated to verify the relationship between the predicted risk and observed frequency (Figure 5). The C-index of the training cohort and validation cohort was 0.913 (95%CI: 0.870–0.957), and 0.840 (95%CI: 0.753–0.927), respectively. And the brier score was 0.077 (95%CI: 0.051–0.103), and 0.106 (95%CI: 0.066–0.146) respectively. These results demonstrated the nomogram model had a good accuracy in predicting postoperative AL in patients with colorectal carcinoma.

### Receiver operating characteristic analysis

**Figure 6:**
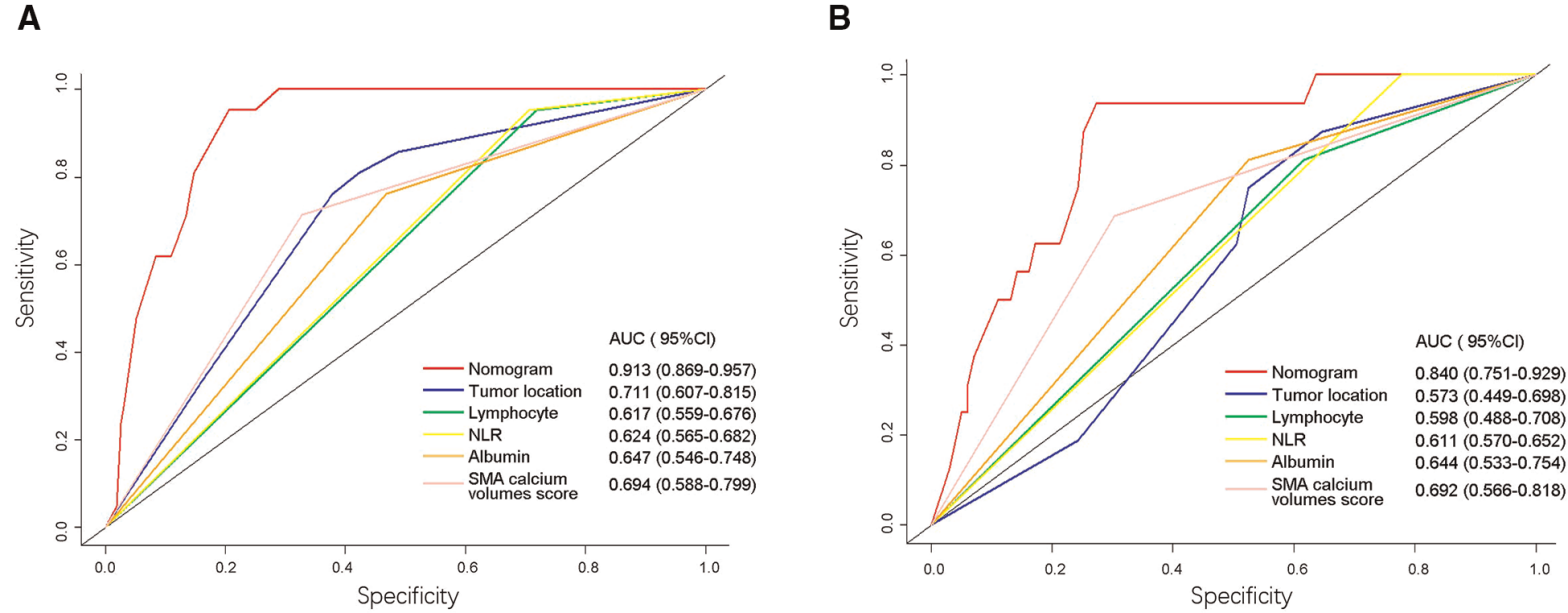
Receiver operating characteristic (ROC) curves of the nomogram and five risk factors in training cohort (**A**) and validation cohort (**B**). The values of AUC (95%CI) for the nomogram and five risk factors were listed in the figure. The *X*-axis represented the specificity of the nomogram and five risk factors to predict AL, and the *Y*-axis represented the sensitivity of these items. NLR, neutrophil-lymphocyte ratio; SMA, superior mesenteric artery; AUC, area under the curve.

We then used ROC analysis to compare the accuracy of the nomogram and five independent risk factors. The AUC for postoperative AL in training cohort and validation cohort were calculated: the nomogram, 0.913 (95%CI: 0.869–0.957) and 0.840 (95%CI: 0.751–0.929); tumor location, 0.711 (95%CI: 0.607–0.815) and 0.573 (95%CI: 0.449–0.698); preoperative lymphocyte, 0.617 (95%CI: 0.559–0.676) and 0.598 (95%CI: 0.448–0.708); preoperative NLR, 0.624 (95%CI: 0.565–0.682) and 0.611 (95%CI: 0.570–0.652); preoperative albumin, 0.647 (95%CI: 0.546–0.748) and 0.644 (95%CI: 0.533–0.754); SMA calcium volumes score, 0.694 (95%CI: 0.588–0.799) and 0.692 (95%CI: 0.566–0.818), respectively (Figure 6).

## Discussion

AL is a serious complication that can affect the short-term quality of life and long-term prognosis after colorectal cancer surgery (5). Surgeons usually judge whether the anastomotic bowel is ischemic by observing the color of the bowel in surgery, but this method is too subjective. Recent studies showed that intraoperative application of indocyanine green, selective ileostomy and other methods could also reduce the morbidity of postoperative AL (7, 9, 23). However, these methods were easily biased by some factors and new evaluation methods were needed. Preoperative malnutrition, serious inflammatory response and abdominal artery calcification might have an adverse effect on postoperative tissue healing (10, 12, 19, 20), and these risk factors were included in this study to construct the nomogram.

The baseline data of 292 patients were retrospectively collected in this study and multivariate analysis showed that tumor location, preoperative albumin, preoperative lymphocyte, preoperative NLR, and SMA calcium volumes score were the risk factors for AL after colorectal cancer surgery. It was worth noting that BMI, brinkman index and sake index were not significantly correlated with the occurrence of AL, which may be attributed to the small sample size and single-center study. Many studies showed that the morbidity of AL was the greatest in the patients after low anterior resection for rectal cancer (12, 24). The patients had higher morbidity of AL when the tumor was closer to the anal margin. This study demonstrated that tumor location was the strongest risk factor for colorectal cancer surgery, and surgeons should pay special attention to the prevention of AL in these patients. The preoperative nutrition of the patients could affect the occurrence of postoperative complications. A multicenter prospective study of 3,193 patients conducted by Matteo et al. showed that low preoperative albumin was an independent risk factor for AL after colon resection for cancer (25). The low albumin usually represented severe malnutrition in cancer patients, and the patients were generally in poor condition with serious symptoms and more malignant tumors. Adequate preoperative nutritional support for such patients was necessary to improve postoperative recovery. The preoperative systemic inflammatory response was significantly associated with postoperative AL, but the mechanism of inflammation in AL was still unclear (10). The preoperative NLR was a better indicator to reflect the basic level of the inflammatory response (18, 26). The preoperative NLR was associated with the occurrence of major postoperative surgical complications (27), and one study showed that the high perioperative lymphocyte and NLR were related to the occurrence of AL in patients with colorectal cancer surgery (28). Another study showed that preoperative NLR was an independent risk factor for postoperative AL of rectal cancer, but its specificity was not high (29). This study concluded that high preoperative lymphocytes and NLR may lead to AL, which might be due to the local continuous infiltration of numerous inflammatory cells, which inhibited angiogenesis and fibroblast growth (17).

The abdominal aorta and its branches are important blood vessels that surgeons should pay special attention to protecting. Some studies have shown that some factors related to vascular abnormalities can affect the outcome of gastrointestinal surgery. The calcification of the thoracic aorta, celiac axis and SMA increased the risk of AL after esophageal cancer surgery (30, 31). One meta-analysis showed that calcification of the abdominal aorta might increase the risk of colorectal AL (19). Some prospective studies that explored the correlation between arterial calcification and AL in patients after colorectal cancer surgery were needed. The results of multivariate analysis in this study showed that a high SMA calcification score was an independent risk factor for AL after colorectal cancer surgery. The occurrence of AL was affected by blood perfusion at the anastomotic site. The blood supply of the proximal bowel always comes from direct or compensatory perfusion of SMA after colorectal cancer surgery. SMA with high calcification score has less blood flow, which may reduce the amount of blood circulation in the intestine and prolong the time of compensatory blood supply. Eventually, intestinal healing may be delayed due to insufficient nutrient supply, and the risk of AL will increase.

In this paper, a nomogram based on the multivariate analysis model was constructed to accurately predict the morbidity of AL after colorectal cancer surgery. The nomogram showed that tumor location and preoperative NLR were the two important risk factors for AL. The AL was most likely to occur in the rectum, sigmoid colon and left-side colon, and surgeons should carefully evaluate these patients' conditions during the perioperative period. The high-level NLR and lymphocyte indicated that the patients had unsuited inflammatory response which might inhibit the tissue growth at the anastomotic site. Many studies showed that some perioperative treatments like NSAIDs did not achieve the purpose of preventing AL (32, 33). Preoperative low albumin and severe SMA calcification could exacerbate nutrient deficiency at the anastomotic site. The severe SMA calcification was also the main risk factor and got approximately 74 points in the nomogram, which suggested that adequate blood supply was necessary for anastomotic healing. Preoperative treatment may be necessary for patients with obvious SMA calcification. The calibration curves in both the training cohort and validation cohort proved the consistency between the predicted value and the actual observation value. The ROC curve of the nomogram had the largest AUC, and the predictive accuracy of the nomogram was better than that of other risk factors. This nomogram can be used clinically to accurately predict the possibility of postoperative AL in patients with colorectal cancer.

Some limitations still existed in this study. This study was a single-center retrospective study, and more samples were needed to be included to obtain high-level evidence. The nomogram in this study showed excellent performance in internal verification, but it still needed to be further tested with more samples.

In conclusion, the occurrence of AL was affected by multiple factors, and a comprehensive evaluation of patients would be necessary. The tumor location closes the anal margin, malnutrition, excessive inflammatory response and severe SMA calcification could increase the risk of AL in patients after colorectal cancer surgery. The nomogram, combining the above risk factors, could effectively predict the probability of postoperative AL in patients with colorectal carcinoma, which can guide surgeons to carry out the appropriate treatment for the prevention of AL.

## Data Availability

The datasets generated during and/or analysed during the current study are available from the corresponding author on reasonable request.

## References

[1] Argilés G Tabernero J Labianca R Hochhauser D Salazar R Iveson T Localised colon cancer: esmo clinical practice guidelines for diagnosis, treatment and follow-up. Ann Oncol. (2020) 31(10):1291–305. 10.1016/j.annonc.2020.06.022 32702383

[2] Glynne-Jones R Wyrwicz L Tiret E Brown G Rödel C Cervantes A Rectal cancer: esmo clinical practice guidelines for diagnosis, treatment and follow-up. Ann Oncol. (2017) 28(suppl_4):iv22–40. 10.1093/annonc/mdx224 28881920

[3] Paun BC Cassie S MacLean AR Dixon E Buie WD. Postoperative complications following surgery for rectal cancer. Ann Surg. (2010) 251(5):807–18. 10.1097/SLA.0b013e3181dae4ed 20395841

[4] Mirnezami A Mirnezami R Chandrakumaran K Sasapu K Sagar P Finan P. Increased local recurrence and reduced survival from colorectal cancer following anastomotic leak: systematic review and meta-analysis. Ann Surg. (2011) 253(5):890–9. 10.1097/SLA.0b013e3182128929 21394013

[5] Krarup P-M Nordholm-Carstensen A Jorgensen LN Harling H. Anastomotic leak increases distant recurrence and long-term mortality after curative resection for colonic cancer: a nationwide cohort study. Ann Surg. (2014) 259(5):930–8. 10.1097/SLA.0b013e3182a6f2fc 24045445

[6] Yang J Chen Q Jindou L Cheng Y. The influence of anastomotic leakage for rectal cancer oncologic outcome: a systematic review and meta-analysis. J Surg Oncol. (2020) 121(8):1283–97. 10.1002/jso.25921 32243581

[7] Shen R Zhang Y Wang T. Indocyanine green fluorescence angiography and the incidence of anastomotic leak after colorectal resection for colorectal cancer: a meta-analysis. Dis Colon Rectum. (2018) 61(10):1228–34. 10.1097/DCR.0000000000001123 30192332

[8] Matthiessen P Hallböök O Rutegård J Simert G Sjödahl R. Defunctioning stoma reduces symptomatic anastomotic leakage after low anterior resection of the Rectum for cancer: a randomized multicenter trial. Ann Surg. (2007) 246(2):207–14. 10.1097/SLA.0b013e3180603024 17667498 PMC1933561

[9] Hüser N Michalski CW Erkan M Schuster T Rosenberg R Kleeff J Systematic review and meta-analysis of the role of defunctioning stoma in low rectal cancer surgery. Ann Surg. (2008) 248(1):52–60. 10.1097/SLA.0b013e318176bf65 18580207

[10] Foppa C Ng SC Montorsi M Spinelli A. Anastomotic leak in colorectal cancer patients: new insights and perspectives. Eur J Surg Oncol. (2020) 46(6):943–54. 10.1016/j.ejso.2020.02.027 32139117

[11] McDermott FD Heeney A Kelly ME Steele RJ Carlson GL Winter DC. Systematic review of preoperative, intraoperative and postoperative risk factors for colorectal anastomotic leaks. Br J Surg. (2015) 102(5):462–79. 10.1002/bjs.9697 25703524

[12] Trencheva K Morrissey KP Wells M Mancuso CA Lee SW Sonoda T Identifying important predictors for anastomotic leak after colon and rectal resection: prospective study on 616 patients. Ann Surg. (2013) 257(1):108–13. 10.1097/SLA.0b013e318262a6cd 22968068

[13] Lee SY Jung MR Kim CH Kim YJ Kim HR. Nutritional risk screening score is an independent predictive factor of anastomotic leakage after rectal cancer surgery. Eur J Clin Nutr. (2018) 72(4):489–95. 10.1038/s41430-018-0112-3 29459787

[14] Xu H Kong F. Malnutrition-related factors increased the risk of anastomotic leak for rectal cancer patients undergoing surgery. Biomed Res Int. (2020) 2020:5059670. 10.1155/2020/5059670 32461995 PMC7212272

[15] Reisinger KW van Vugt JLA Tegels JJW Snijders C Hulsewé KWE Hoofwijk AGM Functional compromise reflected by sarcopenia, frailty, and nutritional depletion predicts adverse postoperative outcome after colorectal cancer surgery. Ann Surg. (2015) 261(2):345–52. 10.1097/SLA.0000000000000628 24651133

[16] Tian W Xu X Yao Z Yang F Huang M Zhao R Early enteral nutrition could reduce risk of recurrent leakage after definitive resection of anastomotic leakage after colorectal cancer surgery. World J Surg. (2021) 45(1):320–30. 10.1007/s00268-020-05787-6 32975647

[17] Hanahan D Weinberg RA. Hallmarks of cancer: the next generation. Cell. (2011) 144(5):646–74. 10.1016/j.cell.2011.02.013 21376230

[18] Tuomisto AE Mäkinen MJ Väyrynen JP. Systemic inflammation in colorectal cancer: underlying factors, effects, and prognostic significance. World J Gastroenterol. (2019) 25(31):4383–404. 10.3748/wjg.v25.i31.4383 31496619 PMC6710177

[19] Tong L Xie D Song X Wu X Wen S Liu A. Is abdominal vascular calcification score valuable in predicting the occurrence of colorectal anastomotic leakage? A meta-analysis. Int J Colorectal Dis. (2020) 35(4):641–53. 10.1007/s00384-020-03513-1 32016599

[20] Postaire B Abet E Montigny P Vent PA. Does the degree of calcification of the celiac trunk and superior mesenteric artery on preoperative computerized tomography predict the risk of anastomotic leak after right colectomy? A single center retrospective study. J Visc Surg. (2019) 156(3):191–5. 10.1016/j.jviscsurg.2018.10.006 30391213

[21] Achenbach S Moselewski F Ropers D Ferencik M Hoffmann U MacNeill B Detection of calcified and noncalcified coronary atherosclerotic plaque by contrast-enhanced, submillimeter multidetector spiral computed tomography: a segment-based comparison with intravascular ultrasound. Circulation. (2004) 109(1):14–7. 10.1161/01.CIR.0000111517.69230.0F 14691045

[22] Harrell FE Califf RM Pryor DB Lee KL Rosati RA. Evaluating the yield of medical tests. JAMA. (1982) 247(18):2543–6. 10.1001/jama.1982.03320430047030 7069920

[23] Montedori A Cirocchi R Farinella E Sciannameo F Abraha I. Covering ileo- or colostomy in anterior resection for rectal carcinoma. Cochrane Database Syst Rev. (2010) 5:CD006878. 10.1002/14651858.CD006878.pub2 PMC12721704 20464746

[24] Park JS Choi G-S Kim SH Kim HR Kim NK Lee KY Multicenter analysis of risk factors for anastomotic leakage after laparoscopic rectal cancer excision: the Korean laparoscopic colorectal surgery study group. Ann Surg. (2013) 257(4):665–71. 10.1097/SLA.0b013e31827b8ed9 23333881

[25] Frasson M Flor-Lorente B Rodríguez JLR Granero-Castro P Hervás D Alvarez Rico MA Risk factors for anastomotic leak after colon resection for cancer: multivariate analysis and nomogram from a multicentric, prospective, national study with 3,193 patients. Ann Surg. (2015) 262(2):321–30. 10.1097/SLA.0000000000000973 25361221

[26] Guthrie GJK Charles KA Roxburgh CSD Horgan PG McMillan DC Clarke SJ. The systemic inflammation-based neutrophil-lymphocyte ratio: experience in patients with cancer. Crit Rev Oncol Hematol. (2013) 88(1):218–30. 10.1016/j.critrevonc.2013.03.010 23602134

[27] Josse JM Cleghorn MC Ramji KM Jiang H Elnahas A Jackson TD The neutrophil-to-lymphocyte ratio predicts Major perioperative complications in patients undergoing colorectal surgery. Colorectal Dis. (2016) 18(7):O236–O42. 10.1111/codi.13373 27154050

[28] Paliogiannis P Deidda S Maslyankov S Paycheva T Farag A Mashhour A Blood cell count indexes as predictors of anastomotic leakage in elective colorectal surgery: a multicenter study on 1,432 patients. World J Surg Oncol. (2020) 18(1):89. 10.1186/s12957-020-01856-1 32375770 PMC7204308

[29] Miyakita H Sadahiro S Saito G Okada K Tanaka A Suzuki T. Risk scores as useful predictors of perioperative complications in patients with rectal cancer who received radical surgery. Int J Clin Oncol. (2017) 22(2):324–31. 10.1007/s10147-016-1054-1 27783239 PMC5378746

[30] Hoek VT Edomskis PP Menon AG Kleinrensink G-J Lagarde SM Lange JF Arterial calcification is a risk factor for anastomotic leakage after esophagectomy: a systematic review and meta-analysis. Eur J Surg Oncol. (2020) 46(11):1975–88. 10.1016/j.ejso.2020.06.019 32883552

[31] Koyanagi K Ozawa S Ninomiya Y Oguma J Kazuno A Yatabe K Association between indocyanine green fluorescence blood flow speed in the gastric conduit wall and superior mesenteric artery calcification: predictive significance for anastomotic leakage after esophagectomy. Esophagus. (2021) 18(2):248–57. 10.1007/s10388-020-00797-8 33165752

[32] Hakkarainen TW Steele SR Bastaworous A Dellinger EP Farrokhi E Farjah F Nonsteroidal anti-inflammatory drugs and the risk for anastomotic failure: a report from Washington state's surgical care and outcomes assessment program (scoap). JAMA Surg. (2015) 150(3):223–8. 10.1001/jamasurg.2014.2239 25607250 PMC4524521

[33] Klein M Gögenur I Rosenberg J. Postoperative use of non-steroidal anti-inflammatory drugs in patients with anastomotic leakage requiring reoperation after colorectal resection: cohort study based on prospective data. Br Med J. (2012) 345:e6166. 10.1136/bmj.e6166 23015299 PMC3458793

